# Supplementary material for: Can open source large language models be used for tumor documentation in Germany?—An evaluation on urological doctors’ notes
Source: BioData Min. 2025 Jul 24;18:48. doi: 10.1186/s13040-025-00463-8 (PMC12291363; doi:10.1186/s13040-025-00463-8)
Supplement: Supplementary file 1 — Supplementary Material 1: Table S1. Relationship between patients, text snippets, and the annotated diagnoses in the evaluation dataset. [file 13040_2025_463_MOESM1_ESM.html]

Table S1: Relationship between patients, text snippets, and annotated diagnoses


## Table S1

Relationship between patients, text snippets, and the annotated diagnoses.
The table columns show the patient identifier, the number of snippets per patient,
the snippet identifier (i.e., the array index in the
Hugging Face dataset), and the diagnosis codes that were annotated for
the snippet.

| Patient ID | Number of snippets | Snippet ID | Diagnoses |
| --- | --- | --- | --- |
| 1 | 5 | 0 | C61 |
|  |  | 1 | C61 |
|  |  | 2 | C61 |
|  |  | 3 | C61 |
|  |  | 4 | C61 |
| 2 | 4 | 5 | C61 |
|  |  | 6 | C61 |
|  |  | 7 | C61 |
|  |  | 8 | C61 |
| 3 | 4 | 9 |  |
|  |  | 10 |  |
|  |  | 11 | C61 |
|  |  | 12 |  |
| 4 | 6 | 13 | C61 |
|  |  | 14 | C61 |
|  |  | 15 | C61 |
|  |  | 16 | C61 |
|  |  | 17 | C61 |
|  |  | 18 | C61 |
| 5 | 7 | 19 |  |
|  |  | 20 |  |
|  |  | 21 | C61 |
|  |  | 22 | C61 |
|  |  | 23 | C61 |
|  |  | 24 |  |
|  |  | 25 |  |
| 6 | 7 | 26 | C61 |
|  |  | 27 | C61 |
|  |  | 28 |  |
|  |  | 29 |  |
|  |  | 30 | C61 |
|  |  | 31 |  |
|  |  | 32 | C61 |
| 7 | 13 | 33 | C61 |
|  |  | 34 | C61 |
|  |  | 35 |  |
|  |  | 36 |  |
|  |  | 37 |  |
|  |  | 38 |  |
|  |  | 39 | C61 |
|  |  | 40 | C61 |
|  |  | 41 | C61 |
|  |  | 42 |  |
|  |  | 43 |  |
|  |  | 44 | C61 |
|  |  | 45 |  |
| 8 | 12 | 46 |  |
|  |  | 47 | C61 |
|  |  | 48 | C61 |
|  |  | 49 |  |
|  |  | 50 |  |
|  |  | 51 |  |
|  |  | 52 |  |
|  |  | 53 |  |
|  |  | 54 |  |
|  |  | 55 |  |
|  |  | 56 |  |
|  |  | 57 | C61 |
| 9 | 7 | 58 | C61C67 |
|  |  | 59 | C61C67 |
|  |  | 60 | C61C67 |
|  |  | 61 | C61C68 |
|  |  | 62 | C61C68 |
|  |  | 63 | C61C67 |
|  |  | 64 |  |
| 10 | 11 | 65 | C61 |
|  |  | 66 | C61 |
|  |  | 67 | C61 |
|  |  | 68 | C61 |
|  |  | 69 | C61 |
|  |  | 70 | C61 |
|  |  | 71 | C61 |
|  |  | 72 | C61 |
|  |  | 73 | C61 |
|  |  | 74 | C61 |
|  |  | 75 | C61 |
| 11 | 3 | 76 | C61C67 |
|  |  | 77 | C61C67 |
|  |  | 78 | C18C61C67 |
| 12 | 6 | 79 | C61 |
|  |  | 80 |  |
|  |  | 81 |  |
|  |  | 82 | C61 |
|  |  | 83 | C61 |
|  |  | 84 |  |
| 13 | 4 | 85 |  |
|  |  | 86 | C61C67 |
|  |  | 87 | C61C67 |
|  |  | 88 | C61C67 |
| 14 | 3 | 89 | C61 |
|  |  | 90 | C61 |
|  |  | 91 | C61 |
| 15 | 10 | 92 | C61 |
|  |  | 93 | C61 |
|  |  | 94 | C61 |
|  |  | 95 | C61 |
|  |  | 96 | C61 |
|  |  | 97 | C61 |
|  |  | 98 |  |
|  |  | 99 | C61 |
|  |  | 100 | C61 |
|  |  | 101 | C61 |
| 16 | 10 | 102 | C09C61C83 |
|  |  | 103 |  |
|  |  | 104 | C09C34C61C83 |
|  |  | 105 | C09C34C61C83 |
|  |  | 106 | C09C34C61C83 |
|  |  | 107 | C09C34C61C83 |
|  |  | 108 | C09C34C61C83 |
|  |  | 109 | C09C34C61C83 |
|  |  | 110 | C09C34C61C83 |
|  |  | 111 | C09C34C61C83 |
| 17 | 6 | 112 | C61 |
|  |  | 113 | C61 |
|  |  | 114 | C61 |
|  |  | 115 | C61 |
|  |  | 116 | C61 |
|  |  | 117 | C61 |
| 18 | 6 | 118 | C61C90 |
|  |  | 119 | C61D47 |
|  |  | 120 | C90 |
|  |  | 121 | C61 |
|  |  | 122 | C90 |
|  |  | 123 | C90 |
| 19 | 4 | 124 | C61 |
|  |  | 125 | C61 |
|  |  | 126 | C61 |
|  |  | 127 | C61 |
| 20 | 4 | 128 | C61 |
|  |  | 129 | C61 |
|  |  | 130 | C61 |
|  |  | 131 | C61 |
| 21 | 4 | 132 |  |
|  |  | 133 | C61 |
|  |  | 134 |  |
|  |  | 135 | C61 |
| 22 | 4 | 136 | C45 |
|  |  | 137 | C45 |
|  |  | 138 | C45 |
|  |  | 139 | C45 |
| 23 | 4 | 140 |  |
|  |  | 141 | C61 |
|  |  | 142 |  |
|  |  | 143 | C61 |
| 24 | 3 | 144 | C67 |
|  |  | 145 | C34C61 |
|  |  | 146 | C34C61C67 |
| 25 | 2 | 147 | C61C67 |
|  |  | 148 | C67 |
