# Supplementary material for: Can open source large language models be used for tumor documentation in Germany?—An evaluation on urological doctors’ notes
Source: BioData Min. 2025 Jul 24;18:48. doi: 10.1186/s13040-025-00463-8 (PMC12291363; doi:10.1186/s13040-025-00463-8)
Supplement: Supplementary file 2 — Supplementary Material 2: Figure S1. Sensitivity, Specificity and Accuracy for detecting a tumor diagnosis in a text snippet using different models. An interactive visualization of the results from Step 1 of the evaluation. [file 13040_2025_463_MOESM2_ESM.html]

Figure S1


# Sensitivity, Specificity and Accuracy for Detecting a Tumor Diagnosis in a Text Snippet Using Different Models Interactive Bar Plot Guide Interactivity Features: - Click on any legend item (prompting variants, metrics, models) to toggle their visibility - Drag and drop model charts to reorder them as needed - Hover over bars to see detailed information Tips: - Hide/show specific models to focus your comparison - Toggle between metrics to analyze different aspects of performance

Prompting Variants:

Fictious examples from urology

 Fictious examples from gynecology

Zero-shot prompting

 Two-shot prompting

 Four-shot prompting

 Six-shot prompting

Metrics:

Sensitivity
Specificity
Accuracy
NA

Models:
