# Supplementary material for: Can open source large language models be used for tumor documentation in Germany?—An evaluation on urological doctors’ notes
Source: BioData Min. 2025 Jul 24;18:48. doi: 10.1186/s13040-025-00463-8 (PMC12291363; doi:10.1186/s13040-025-00463-8)
Supplement: Supplementary file 3 — Supplementary Material 3: Figure S2. Proportion of correctly mapped ICD-10 codes using different models. An interactive visualization of the results from Step 2 of the evaluation. [file 13040_2025_463_MOESM3_ESM.html]

Figure S2


# Proportion of Correctly Mapped ICD-10 Codes Using Different Models Interactive Bar Plot Guide Interactivity Features: - Click on any legend item (prompting variants, metrics, models) to toggle their visibility - Drag and drop model charts to reorder them as needed - Hover over bars to see detailed information Tips: - Hide/show specific models to focus your comparison - Toggle between metrics to analyze different aspects of performance

Prompting Variants:

Fictious examples from urology


Fictious examples from gynecology

Zero-shot prompting with two ICD-10 codes as examples


Zero-shot prompting with two examples + text context


Two-shot prompting with two ICD-10 codes as examples


Two-shot prompting with two examples + text context

Metrics:

All diagnoses found
No incorrect diagnosis
Snippet correct

Models:
