# Supplementary material for: Can open source large language models be used for tumor documentation in Germany?—An evaluation on urological doctors’ notes
Source: BioData Min. 2025 Jul 24;18:48. doi: 10.1186/s13040-025-00463-8 (PMC12291363; doi:10.1186/s13040-025-00463-8)
Supplement: Supplementary file 4 — Supplementary Material 4: Figure S3. Proportion of correctly identified first diagnosis dates using different models. An interactive visualization of the results from Step 3 of the evaluation. [file 13040_2025_463_MOESM4_ESM.html]

Figure S3


# Proportion of Correctly Identified First Diagnosis Dates Using Different Models Interactive Bar Plot Guide Interactivity Features: - Click on any legend item (prompting variants, metrics, models) to toggle their visibility - Drag and drop model charts to reorder them as needed - Hover over bars to see detailed information Tips: - Hide/show specific models to focus your comparison - Toggle between metrics to analyze different aspects of performance

Prompting Variants:

Zero-shot prompting

 Zero-shot prompting using dates filtered with regular expressions

 Three-shot prompting with three fictious examples

 Three-shot prompting using dates filtered with regular expressions

Filter dates and verify using zero-shot prompting

 Filter dates and verify using four-shot prompting

Metrics:

Accuracy (excl. NA)
Accuracy (NA=wrong)
NA

Models:
