# Supplementary material for: Can open source large language models be used for tumor documentation in Germany?—An evaluation on urological doctors’ notes
Source: BioData Min. 2025 Jul 24;18:48. doi: 10.1186/s13040-025-00463-8 (PMC12291363; doi:10.1186/s13040-025-00463-8)
Supplement: Supplementary file 5 — Supplementary Material 5: Appendix with detailed result tables (Appendix A) and the prompt templates (Appendix B). [file 13040_2025_463_MOESM5_ESM.pdf]

Appendix for article “Can open source large language models be used for tumor documentation in Germany?—An evaluation on urological doctors’ notes”

Content

Appendix A: Detailed result tables 2
Appendix B: Prompt templates (in German) 6
Prompt templates for Step 1 6
Prompt templates for Step 2 13
Prompt templates for Step 3 17

Appendix A: Detailed result tables

Table A-1 Results of Step 1: Performance of different models for detecting a tumor diagnosis in a text. Unusable answers ("NA") count as wrong answers for calculating the accuracy here. These values are ignored when calculating sensitivity and specificity. The column headers indicate the prompt type: the number represents the count of examples, and the suffix specifies whether the examples come from a fictitious urology unit or a fictitious gynecological unit.

		**0**	**2-gyn**	**2-uro**	**4-gyn**	**4-uro**	**6-gyn**	**6-uro**
BioMistral-7B	Accuracy (NA=wrong)	0.49	0.77	0.75	0.70	0.79	0.58	0.64
	NA	0.37	0.15	0.17	0.15	0.15	0.32	0.28
	Sensitivity	1.00	0.96	0.91	0.84	0.93	0.87	0.91
	Specificity	0.12	0.77	0.91	0.77	0.91	0.81	0.79
EuroLLM 1.7B	Accuracy (NA=wrong)	0.12	0.38	0.46	0.44	0.56	0.34	0.34
	NA	0.79	0.03	0.04	0.03	0.07	0.03	0.01
	Sensitivity	1.00	0.27	0.46	0.37	0.68	0.15	0.12
	Specificity	0.00	0.80	0.53	0.69	0.38	0.97	1.00
LLama 3.2 1B	Accuracy (NA=wrong)	0.21	0.41	0.38	0.41	0.39	0.19	0.27
	NA	0.14	0.26	0.15	0.17	0.18	0.27	0.09
	Sensitivity	0.00	0.41	0.27	0.35	0.33	0.07	0.08
	Specificity	0.97	0.86	0.89	0.86	0.85	0.96	0.89
LLama 3.2 3B	Accuracy (NA=wrong)	0.17	0.91	0.91	0.89	0.91	0.72	0.94
	NA	0.81	0.00	0.03	0.00	0.01	0.00	0.00
	Sensitivity	0.96	0.89	0.94	0.85	0.94	0.63	0.96
	Specificity	0.80	0.97	0.92	1.00	0.83	1.00	0.89
LeoLM 7B Chat	Accuracy (NA=wrong)	0.38	0.58	0.62	0.59	0.60	0.58	0.70
	NA	0.52	0.22	0.19	0.26	0.28	0.36	0.22
	Sensitivity	1.00	0.99	0.93	1.00	1.00	0.99	1.00
	Specificity	0.00	0.09	0.28	0.23	0.36	0.55	0.60
Llama 3.1 70B	Accuracy (NA=wrong)	0.35	0.83	0.86	0.83	0.87	0.85	0.88
	NA	0.18	0.01	0.01	0.02	0.01	0.01	0.01
	Sensitivity	0.23	0.82	0.88	0.85	0.88	0.88	0.89
	Specificity	0.97	0.86	0.81	0.83	0.84	0.81	0.89
Llama 3.1 8B	Accuracy (NA=wrong)	0.74	0.91	0.96	0.93	0.98	0.90	0.98
	NA	0.18	0.01	0.00	0.00	0.00	0.01	0.00
	Sensitivity	1.00	0.91	0.98	0.91	1.00	0.89	0.99
	Specificity	0.43	0.95	0.89	0.97	0.92	0.97	0.95
Llama 3.1 SauerkrautLM 8B	Accuracy (NA=wrong)	0.87	0.95	0.95	0.95	0.96	0.93	0.97
	NA	0.06	0.02	0.01	0.01	0.01	0.01	0.02
	Sensitivity	1.00	0.96	1.00	0.95	1.00	0.91	1.00
	Specificity	0.73	0.97	0.83	0.97	0.89	1.00	0.94

Mistral 7B v0.3	Accuracy (NA=wrong)	0.76	0.92	0.93	0.91	0.91	0.89	0.91
	NA	0.11	0.00	0.00	0.03	0.00	0.05	0.00
	Sensitivity	0.99	0.94	0.99	0.94	0.96	0.94	0.96
	Specificity	0.31	0.86	0.73	0.89	0.76	0.92	0.76
Mistral NeMo 12B	Accuracy (NA=wrong)	0.82	0.93	0.93	0.93	0.93	0.93	0.94
	NA	0.14	0.00	0.00	0.00	0.00	0.01	0.00
	Sensitivity	0.97	1.00	1.00	0.95	0.99	0.94	1.00
	Specificity	0.92	0.70	0.70	0.89	0.76	0.95	0.76
Mixtral 8x7B	Accuracy (NA=wrong)	0.81	0.94	0.92	0.89	0.91	0.84	0.93
	NA	0.13	0.01	0.03	0.03	0.04	0.07	0.04
	Sensitivity	0.92	0.95	0.96	0.91	0.96	0.87	0.97
	Specificity	0.95	0.97	0.92	0.97	0.92	0.97	0.97

Table A-2 Results of Step 2: Proportion of snippets with correctly mapped ICD-10 codes for the different models and prompt types. The diagnosis labels from Step 1 are used in the prompts. The rows with only the names of the models in the first column use the results from the same model specified and the best prompt type for this model from Step 1. Additionally, the values using the best model/prompt combination overall from Step 1 are reported in the table. The column headers specify the prompt type: The number represents the number of examples, the suffix "uro"/"gyn" whether the prompt is tailed for urological diagnoses or diagnoses from a fictitious gynecological unit, and the suffix "ctx" indicates whether the full snippet has been added as context to the prompt.

	**0-uro**	**0-gyn**	**0-uro-ctx**	**0-gyn-ctx**	**2-uro**	**2-gyn**	**2-uro-ctx**	**2-gyn-ctx**
BioMistral-7B	0.86	0.73	0.86	0.83	0.84	0.80	0.84	0.80
BioMistral-7B - using results from best model in Step 1	0.82	0.73	0.87	0.82	0.77	0.78	0.78	0.73
EuroLLM 1.7B	0.52	0.02	0.57	0.01	0.18	0.03	0.08	0.07
EuroLLM 1.7B - using results from best model in Step 1	0.73	0.00	0.75	0.02	0.08	0.02	0.04	0.05
LLama 3.2 1B	0.06	0.00	0.50	0.03	0.36	0.25	0.50	0.06
LLama 3.2 1B - using results from best model in Step 1	0.27	0.05	0.70	0.09	0.70	0.27	0.61	0.19
LLama 3.2 3B	0.73	0.63	0.77	0.65	0.64	0.64	0.66	0.68
LLama 3.2 3B - using results from best model in Step 1	0.65	0.50	0.77	0.59	0.58	0.50	0.58	0.58
LeoLM 7B Chat	0.54	0.39	0.43	0.28	0.62	0.60	0.48	0.53
LeoLM 7B Chat - using results from best model in Step 1	0.53	0.27	0.41	0.26	0.59	0.51	0.41	0.39

Llama 3.1 70B	0.71	0.66	0.77	0.63	0.53	0.57	0.75	0.75
Llama 3.1 70B - using results from best model in Step 1	0.77	0.73	0.81	0.73	0.60	0.69	0.82	0.77
Llama 3.1 8B (the best model in Step 1)	0.88	0.70	0.88	0.81	0.71	0.79	0.78	0.81
Llama 3.1 SauerkrautLM 8B	0.88	0.75	0.88	0.87	0.60	0.58	0.70	0.79
Llama 3.1 SauerkrautLM 8B - using results from best model in Step 1	0.88	0.74	0.88	0.85	0.57	0.53	0.69	0.75
Mistral 7B v0.3	0.78	0.71	0.82	0.79	0.75	0.74	0.62	0.69
Mistral 7B v0.3 - using results from best model in Step 1	0.84	0.69	0.88	0.88	0.82	0.81	0.65	0.67
Mistral NeMo 12B	0.86	0.85	0.84	0.83	0.79	0.82	0.81	0.80
Mistral NeMo 12B - using results from best model in Step 1	0.86	0.86	0.86	0.86	0.81	0.86	0.82	0.79
Mixtral 8x7B	0.89	0.85	0.89	0.87	0.81	0.87	0.84	0.88
Mixtral 8x7B - using results from best model in Step 1	0.82	0.76	0.89	0.88	0.73	0.81	0.78	0.88

Table A-3 Results of Step 3 for the different LLM models: Accuracy of finding the first diagnosis dates of the tumors. Unusable answers ("NA") count as wrong answers for calculating the accuracy here. The column headers indicate the prompt type: The number represents the number of examples, the suffix "dates" whether a selection of regex-extracted dates have been presented to the model in the prompt, and the suffix "verify" indicates the variant of Step 3 where the model is asked to verify single possible dates as first diagnosis dates.

	**0**	**0-dates**	**3**	**3-dates**	**0-verify**	**4-verify**
BioMistral-7B	0.20	0.37	0.68	0.44	0.41	0.43
BioMistral-7B - using results from best models in previous steps	0.25	0.35	0.62	0.43	0.49	0.45
EuroLLM 1.7B	0.52	0.52	0.17	0.38	0.51	0.36
EuroLLM 1.7B - using results from best models in previous steps	0.51	0.51	0.21	0.25	0.49	0.53
LLama 3.2 1B	0.46	0.42	0.41	0.42	0.41	0.41
LLama 3.2 1B - using results from best models in previous steps	0.40	0.41	0.43	0.36	0.47	0.47
LLama 3.2 3B	0.26	0.46	0.50	0.61	0.63	0.56
LLama 3.2 3B - using results from best models in previous steps	0.35	0.55	0.58	0.54	0.64	0.56
LeoLM 7B Chat	0.63	0.12	0.57	0.51	0.47	0.50

LeoLM 7B Chat - using results from best models in previous steps	0.53	0.08	0.49	0.51	0.44	0.42
Llama 3.1 70B	0.31	0.59	0.48	0.73	0.43	0.59
Llama 3.1 70B - using results from best models in previous steps	0.43	0.65	0.60	0.84	0.51	0.70
Llama 3.1 8B	0.59	0.71	0.78	0.74	0.81	0.79
Llama 3.1 8B - using results from best models in previous steps	0.58	0.70	0.78	0.74	0.81	0.79
Llama 3.1 SauerkrautLM 8B	0.61	0.56	0.73	0.71	0.58	0.79
Llama 3.1 SauerkrautLM 8B - using results from best models in previous steps	0.65	0.60	0.75	0.73	0.59	0.81
Mistral 7B v0.3	0.53	0.58	0.72	0.71	0.51	0.63
Mistral 7B v0.3 - using results from best models in previous steps	0.54	0.60	0.75	0.73	0.52	0.67
Mistral NeMo 12B	0.88	0.73	0.74	0.72	0.77	0.63
Mistral NeMo 12B - using results from best models in previous steps	0.92	0.74	0.74	0.71	0.78	0.60
Mixtral 8x7B (the best model in Step 2)	0.79	0.73	0.75	0.78	0.70	0.76

Appendix B: Prompt templates (in German)

Prompt templates for Step 1

**Prompt template "0"**
**User:** Gibt es eine oder mehrere Tumordiagnosen in folgendem Diagnosetext? Als Tumordiagnose zählen hier Diagnosen, die im Kapitel II (Neubildungen/neoplasms) der International Classification of Diseases 10th revision (ICD-10) in den Kategorien C00-D48 beschrieben sind. Eine Tumorerkrankung in diesem Sinn ist eine Neubildung abnormen Gewebes aus körpereigenen Zellen im Körper des Patienten. Es ist entscheidend, nur Diagnosen als Tumordiagnosen zu werten, bei denen klar eine solche Tumorerkrankung beschrieben wird. Aussagen über Symptome, Therapien oder andere Erkrankungen sollten nicht als Tumordiagnosen interpretiert werden. Denken Sie nach, ob es sich bei den Diagnosen wirklich um Tumordiagnosen handelt und geben Sie nur Tumordiagnosen zurück, keine anderen Diagnosen. Antworten Sie mit einem JSON-Array, das die Tumordiagnosen als Strings beinhaltet oder mit einem leeren Array, falls es keine eindeutigen Tumordiagnosen gibt. Geben Sie im Antwort-Array keine Diagnosen an, die keine Tumordiagnosen sind.<br>Gibt es eine oder mehrere Tumordiagnosen in folgendem Diagnosetext? Diagnosetext: [SNIPPET-TEXT]

**Prompt template "2-gyn"**
**User:** Gibt es eine oder mehrere Tumordiagnosen in folgendem Diagnosetext? Als Tumordiagnose zählen hier Diagnosen, die im Kapitel II (Neubildungen/neoplasms) der International Classification of Diseases 10th revision (ICD-10) in den Kategorien C00-D48 beschrieben sind. Eine Tumorerkrankung in diesem Sinn ist eine Neubildung abnormen Gewebes aus körpereigenen Zellen im Körper des Patienten. Es ist entscheidend, nur Diagnosen als Tumordiagnosen zu werten, bei denen klar eine solche Tumorerkrankung beschrieben wird. Aussagen über Symptome, Therapien oder andere Erkrankungen sollten nicht als Tumordiagnosen interpretiert werden. Denken Sie nach, ob es sich bei den Diagnosen wirklich um Tumordiagnosen handelt und geben Sie nur Tumordiagnosen zurück, keine anderen Diagnosen. Antworten Sie mit einem JSON-Array, das die Tumordiagnosen als Strings beinhaltet oder mit einem leeren Array, falls es keine eindeutigen Tumordiagnosen gibt. Geben Sie im Antwort-Array keine Diagnosen an, die keine Tumordiagnosen sind.
**Assistant:** Ich werde auf die folgenden Diagnosetexte kurz mit einem JSON-Array antworten und nur Tumordiagnosen berücksichtigen.

User: Gibt es eine oder mehrere Tumordiagnosen in folgendem Diagnosetext?
Diagnosetext: 2005: Invasives lobuläres Mammakarzinom links, Stadium II
Sekundärbefunde: Verdacht auf axilläre Lymphknotenmetastasen links
BRCA1-Genmutation positiv

Assistant: ["Mammakarzinom"]

User: Gibt es eine oder mehrere Tumordiagnosen in folgendem Diagnosetext?
Diagnosetext: CA-125-Wert im Normbereich, unauffällige Sonographie. Kein Anhalt für ein Ovarialkarzinoms.

Assistant: [] (Der Verdacht auf eine erwähnte Tumorerkrankung, hier "Ovarialkarzinom", wird nicht bestätigt.)

User: Gibt es eine oder mehrere Tumordiagnosen in folgendem Diagnosetext?
Diagnosetext: [SNIPPET-TEXT]

Prompt template "2-uro"

User: Gibt es eine oder mehrere Tumordiagnosen in folgendem Diagnosetext? Als Tumordiagnose zählen hier Diagnosen, die im Kapitel II (Neubildungen/neoplasms) der International Classification of Diseases 10th revision (ICD-10) in den Kategorien C00-D48 beschrieben sind. Eine Tumorerkrankung in diesem Sinn ist eine Neubildung abnormen Gewebes aus körpereigenen Zellen im Körper des Patienten. Es ist entscheidend, nur Diagnosen als Tumordiagnosen zu werten, bei denen klar eine solche Tumorerkrankung beschrieben wird. Aussagen über Symptome, Therapien oder andere Erkrankungen sollten nicht als Tumordiagnosen interpretiert werden. Denken Sie nach, ob es sich bei den Diagnosen wirklich um Tumordiagnosen handelt und geben Sie nur Tumordiagnosen zurück, keine anderen Diagnosen. Antworten Sie mit einem JSON-Array, das die Tumordiagnosen als Strings beinhaltet oder mit einem leeren Array, falls es keine eindeutigen Tumordiagnosen gibt. Geben Sie im Antwort-Array keine Diagnosen an, die keine Tumordiagnosen sind.

Assistant: Ich werde auf die folgenden Diagnosetexte kurz mit einem JSON-Array antworten und nur Tumordiagnosen berücksichtigen.

User: Gibt es eine oder mehrere Tumordiagnosen in folgendem Diagnosetext?
Diagnosetext: Prostata-Karzinom pT1. Ausschluss Lebermetastasen

Assistant: ["Prostata-Karzinom"]

User: Gibt es eine oder mehrere Tumordiagnosen in folgendem Diagnosetext?
Diagnosetext: Harnstauung links. Ausschluss eines Urothelkarzinoms

Assistant: [] (Das Urothelkarzinom wird ausgeschlossen.)

User: Gibt es eine oder mehrere Tumordiagnosen in folgendem Diagnosetext?
Diagnosetext: [SNIPPET-TEXT]

Prompt template "4-gyn"

User: Gibt es eine oder mehrere Tumordiagnosen in folgendem Diagnosetext? Als Tumordiagnose zählen hier Diagnosen, die im Kapitel II (Neubildungen/neoplasms) der International Classification of Diseases 10th revision (ICD-10) in den Kategorien C00-D48 beschrieben sind. Eine Tumorerkrankung in diesem Sinn ist eine Neubildung abnormen Gewebes aus körpereigenen Zellen im Körper des Patienten. Es ist entscheidend, nur Diagnosen als Tumordiagnosen zu werten, bei denen klar eine solche Tumorerkrankung beschrieben wird. Aussagen über Symptome, Therapien oder andere Erkrankungen sollten nicht als Tumordiagnosen interpretiert werden. Denken Sie nach, ob es sich bei den Diagnosen wirklich um Tumordiagnosen handelt und geben Sie nur Tumordiagnosen zurück, keine anderen Diagnosen. Antworten Sie mit einem JSON-Array, das die Tumordiagnosen als Strings beinhaltet oder mit einem leeren Array, falls es keine eindeutigen Tumordiagnosen gibt. Geben Sie im Antwort-Array keine Diagnosen an, die keine Tumordiagnosen sind.

Assistant: Ich werde auf die folgenden Diagnosetexte kurz mit einem JSON-Array antworten und nur Tumordiagnosen berücksichtigen.

User: Gibt es eine oder mehrere Tumordiagnosen in folgendem Diagnosetext?
Diagnosetext: 2005: Invasives lobuläres Mammakarzinom links, Stadium II
Sekundärbefunde: Verdacht auf axilläre Lymphknotenmetastasen links
BRCA1-Genmutation positiv

Assistant: ["Mammakarzinom"]

User: Gibt es eine oder mehrere Tumordiagnosen in folgendem Diagnosetext?
Diagnosetext: Die Patientin berichtet über tastbare Veränderung in der rechten Brust.
Die durchgeführte Mammographie und der Brustultraschall zeigen keine Anomalien,
und die Veränderung entspricht einer gutartigen zystischen Läsion.
MRT der Brust bestätigt das Fehlen maligner Merkmale.
Ausschluss eines Mammakarzinoms

**Assistant:** [] (Mammakarzinom wird ausgeschlossen)
**User:** Gibt es eine oder mehrere Tumordiagnosen in folgendem Diagnosetext? Diagnosetext: CA-125-Wert im Normbereich, unauffällige Sonographie. Kein Anhalt für ein Ovarialkarzinoms.
**Assistant:** [] (Die Tumorerkrankung "Ovarialkarzinom" wird nicht bestätigt.)
**User:** Gibt es eine oder mehrere Tumordiagnosen in folgendem Diagnosetext? Diagnosetext: Seröses Ovarialkarzinom des linken Ovars, keine Anzeichen für Fernmetastasen
**Assistant:** ["Ovarialkarzinom"]
**User:** Gibt es eine oder mehrere Tumordiagnosen in folgendem Diagnosetext? Diagnosetext: [SNIPPET-TEXT]

**Prompt template "4-uro"**
**User:** Gibt es eine oder mehrere Tumordiagnosen in folgendem Diagnosetext? Als Tumordiagnose zählen hier Diagnosen, die im Kapitel II (Neubildungen/neoplasms) der International Classification of Diseases 10th revision (ICD-10) in den Kategorien C00-D48 beschrieben sind. Eine Tumorerkrankung in diesem Sinn ist eine Neubildung abnormen Gewebes aus körpereigenen Zellen im Körper des Patienten. Es ist entscheidend, nur Diagnosen als Tumordiagnosen zu werten, bei denen klar eine solche Tumorerkrankung beschrieben wird. Aussagen über Symptome, Therapien oder andere Erkrankungen sollten nicht als Tumordiagnosen interpretiert werden. Denken Sie nach, ob es sich bei den Diagnosen wirklich um Tumordiagnosen handelt und geben Sie nur Tumordiagnosen zurück, keine anderen Diagnosen. Antworten Sie mit einem JSON-Array, das die Tumordiagnosen als Strings beinhaltet oder mit einem leeren Array, falls es keine eindeutigen Tumordiagnosen gibt. Geben Sie im Antwort-Array keine Diagnosen an, die keine Tumordiagnosen sind.
**Assistant:** Ich werde auf die folgenden Diagnosetexte kurz mit einem JSON-Array antworten und nur Tumordiagnosen berücksichtigen.
**User:** Gibt es eine oder mehrere Tumordiagnosen in folgendem Diagnosetext? Diagnosetext: Prostata-Karzinom pT1. Ausschluss Lebermetastasen
**Assistant:** ["Prostata-Karzinom"]

User: Gibt es eine oder mehrere Tumordiagnosen in folgendem Diagnosetext? Diagnosetext: Miktionsbeschwerden. Ausschluss PCA. Z.n. Nierentransplantation 1980 links

Assistant: [] (Die Diagnose Prostatakarzinom/PCA ist hier ausgeschlossen.)

User: Gibt es eine oder mehrere Tumordiagnosen in folgendem Diagnosetext? Diagnosetext: Harnstauung links. Ausschluss eines Urothelkarzinoms

Assistant: [] (Das Urothelkarzinom wird ausgeschlossen.)

User: Gibt es eine oder mehrere Tumordiagnosen in folgendem Diagnosetext? Diagnosetext: Z.n. TUR-B-NR mit dem Nachweis eines Urothelkarzinoms der Harnblase pT2G3

Assistant: ["Urothelkarzinom der Harnblase"]

User: Gibt es eine oder mehrere Tumordiagnosen in folgendem Diagnosetext? Diagnosetext: [SNIPPET-TEXT]

Prompt template "6-gyn"

User: Gibt es eine oder mehrere Tumordiagnosen in folgendem Diagnosetext? Als Tumordiagnose zählen hier Diagnosen, die im Kapitel II (Neubildungen/neoplasms) der International Classification of Diseases 10th revision (ICD-10) in den Kategorien C00-D48 beschrieben sind. Eine Tumorerkrankung in diesem Sinn ist eine Neubildung abnormen Gewebes aus körpereigenen Zellen im Körper des Patienten. Es ist entscheidend, nur Diagnosen als Tumordiagnosen zu werten, bei denen klar eine solche Tumorerkrankung beschrieben wird. Aussagen über Symptome, Therapien oder andere Erkrankungen sollten nicht als Tumordiagnosen interpretiert werden. Denken Sie nach, ob es sich bei den Diagnosen wirklich um Tumordiagnosen handelt und geben Sie nur Tumordiagnosen zurück, keine anderen Diagnosen. Antworten Sie mit einem JSON-Array, das die Tumordiagnosen als Strings beinhaltet oder mit einem leeren Array, falls es keine eindeutigen Tumordiagnosen gibt. Geben Sie im Antwort-Array keine Diagnosen an, die keine Tumordiagnosen sind.

Assistant: Ich werde auf die folgenden Diagnosetexte kurz mit einem JSON-Array antworten und nur Tumordiagnosen berücksichtigen.

User: Gibt es eine oder mehrere Tumordiagnosen in folgendem Diagnosetext? Diagnosetext: 2005: Invasives lobuläres Mammakarzinom links, Stadium II

Sekundärbefunde: Verdacht auf axilläre Lymphknotenmetastasen links<br>BRCA1-Genmutation positiv
**Assistant:** ["Mammakarzinom"]
**User:** Gibt es eine oder mehrere Tumordiagnosen in folgendem Diagnosetext?<br>Diagnosetext: Die Patientin berichtet über tastbare Veränderung in der rechten Brust.<br>Die durchgeführte Mammographie und der Brustultraschall zeigen keine Anomalien,<br>und die Veränderung entspricht einer gutartigen zystischen Läsion.<br>MRT der Brust bestätigt das Fehlen maligner Merkmale.<br>Ausschluss eines Mammakarzinoms
**Assistant:** [] (Mammakarzinom wird ausgeschlossen)
**User:** Gibt es eine oder mehrere Tumordiagnosen in folgendem Diagnosetext?<br>Diagnosetext: CA-125-Wert im Normbereich, unauffällige Sonographie. Kein Anhalt für ein Ovarialkarzinoms.
**Assistant:** [] (Die Tumorerkrankung "Ovarialkarzinom" wird nicht bestätigt.)
**User:** Gibt es eine oder mehrere Tumordiagnosen in folgendem Diagnosetext?<br>Diagnosetext: Seröses Ovarialkarzinom des linken Ovars, keine Anzeichen für Fernmetastasen
**Assistant:** ["Ovarialkarzinom"]
**User:** Gibt es eine oder mehrere Tumordiagnosen in folgendem Diagnosetext?<br>Diagnosetext: Ultraschall der Brust zeigt ein dichtes, aber homogenes Brustgewebe mit mehreren kleinen, nicht-suspekten Zysten.<br>Kein Anhalt für Malignität.<br>Diagnose: Fibrozystische Mastopathie.
**Assistant:** [] (Es wurde keine Tumorerkrankung bei der Patientin festgestellt.<br>Die hier gestellte Diagnose "Fibrozystische Mastopathie" (ICD-10-Code N60) zählt nicht als Tumordiagnose.)
**User:** Gibt es eine oder mehrere Tumordiagnosen in folgendem Diagnosetext?<br>Diagnosetext: Duktales In-situ-Karzinom der rechten Brust.<br>Keine Anzeichen für Fernmetastasen.<br>Hormonrezeptorstatus zeigt Östrogenrezeptor-Positivität. HER2/neu-Status negativ.

Assistant: ["Duktales In-situ-Karzinom der rechten Brust"]

User: Gibt es eine oder mehrere Tumordiagnosen in folgendem Diagnosetext?
Diagnosetext: [SNIPPET-TEXT]

Prompt template "6-uro"

User: Gibt es eine oder mehrere Tumordiagnosen in folgendem Diagnosetext? Als Tumordiagnose zählen hier Diagnosen, die im Kapitel II (Neubildungen/neoplasms) der International Classification of Diseases 10th revision (ICD-10) in den Kategorien C00-D48 beschrieben sind. Eine Tumorerkrankung in diesem Sinn ist eine Neubildung abnormen Gewebes aus körpereigenen Zellen im Körper des Patienten. Es ist entscheidend, nur Diagnosen als Tumordiagnosen zu werten, bei denen klar eine solche Tumorerkrankung beschrieben wird. Aussagen über Symptome, Therapien oder andere Erkrankungen sollten nicht als Tumordiagnosen interpretiert werden. Denken Sie nach, ob es sich bei den Diagnosen wirklich um Tumordiagnosen handelt und geben Sie nur Tumordiagnosen zurück, keine anderen Diagnosen. Antworten Sie mit einem JSON-Array, das die Tumordiagnosen als Strings beinhaltet oder mit einem leeren Array, falls es keine eindeutigen Tumordiagnosen gibt. Geben Sie im Antwort-Array keine Diagnosen an, die keine Tumordiagnosen sind.

Assistant: Ich werde auf die folgenden Diagnosetexte kurz mit einem JSON-Array antworten und nur Tumordiagnosen berücksichtigen.

User: Gibt es eine oder mehrere Tumordiagnosen in folgendem Diagnosetext?
Diagnosetext: Prostata-Karzinom pT1. Ausschluss Lebermetastasen

Assistant: ["Prostata-Karzinom"]

User: Gibt es eine oder mehrere Tumordiagnosen in folgendem Diagnosetext?
Diagnosetext: Miktionsbeschwerden. Ausschluss PCA. Z.n. Nierentransplantation 1980 links

Assistant: [] (Die Diagnose Prostatakarzinom/PCA ist hier ausgeschlossen.)

User: Gibt es eine oder mehrere Tumordiagnosen in folgendem Diagnosetext?
Diagnosetext: Harnstauung links. Ausschluss eines Urothelkarzinoms

Assistant: [] (Das Urothelkarzinom wird ausgeschlossen.)

**User:** Gibt es eine oder mehrere Tumordiagnosen in folgendem Diagnosetext?<br>Diagnosetext: Z.n. TUR-B-NR mit dem Nachweis eines Urothelkarzinoms der Harnblase pT2G3
**Assistant:** ["Urothelkarzinom der Harnblase"]
**User:** Gibt es eine oder mehrere Tumordiagnosen in folgendem Diagnosetext?<br>Diagnosetext: Digital-rektale Untersuchung zeigt eine vergrößerte, aber gleichmäßig geformte Prostata.<br>PSA-Wert im Normbereich. Diagnose: Benigne Prostatahyperplasie (BPH) ohne Hinweise auf Prostatakarzinom.
**Assistant:** [] (Der Verdacht auf die erwähnte Tumorerkrankung, hier "Prostatakarzinom", wird nicht bestätigt.<br>Die Diagnose "Benigne Prostatahyperplasie" (ICD-10-Code N40) zählt nicht als Tumordiagnose.)
**User:** Gibt es eine oder mehrere Tumordiagnosen in folgendem Diagnosetext?<br>Diagnosetext: Z.n. Adenomkarzinom der Prostata pT2b. ED 2015.
**Assistant:** ["Adenomkarzinom der Prostata"]
**User:** Gibt es eine oder mehrere Tumordiagnosen in folgendem Diagnosetext?<br>Diagnosetext: [SNIPPET-TEXT]

Prompt templates for Step 2

**Prompt template "0-gyn-ctx"**
**User:** [Diagnosetext]:<br>[SNIPPET-TEXT]<br>[Ende des Diagnosetexts]<br>Sie haben eine Diagnose im Diagnosetext identifiziert: "[DIAGNOSIS-LABEL]" Was ist der ICD-10-Code für diese Diagnose? Antworten Sie nur kurz mit dem 3-stelligen ICD-10-Code. Wenn es sich bei der Diagnose "[DIAGNOSIS-LABEL]" um ein Mammakarzinom handelt, antworten Sie zum Beispiel mit "C50", bei einem Ovarialkarzinom mit "C56". Die Metastasierung muss bei der Kodierung nicht berücksichtigt werden, das heißt die Codes C77, C78 und C79 für

sekundäre Neubildungen sollten nicht verwendet werden. Die Antwort sollte also niemals "C77", "C78" oder "C79" sein.

Prompt template "0-gyn"

User: Was ist der ICD-10-Code für die Diagnose "[DIAGNOSIS-LABEL]"? Antworten Sie nur kurz mit dem 3-stelligen ICD-10-Code. Wenn es sich bei der Diagnose "[DIAGNOSIS-LABEL]" um ein Mammakarzinom handelt, antworten Sie zum Beispiel mit "C50", bei einem Ovarialkarzinom mit "C56". Die Metastasierung muss bei der Kodierung nicht berücksichtigt werden, das heißt die Codes C77, C78 und C79 für sekundäre Neubildungen sollten nicht verwendet werden. Die Antwort sollte also niemals "C77", "C78" oder "C79" sein.

Prompt template "0-uro-ctx"

User: [Diagnosetext]:
[SNIPPET-TEXT]
[Ende des Diagnosetexts]
Sie haben eine Diagnose im Diagnosetext identifiziert: "[DIAGNOSIS-LABEL]" Was ist der ICD-10-Code für diese Diagnose? Antworten Sie nur kurz mit dem 3-stelligen ICD-10-Code. Wenn es sich bei der Diagnose "[DIAGNOSIS-LABEL]" um ein Prostatakarzinom handelt, antworten Sie zum Beispiel mit "C61", bei einem Urothelkarzinom der Harnblase mit "C67". Die Metastasierung muss bei der Kodierung nicht berücksichtigt werden, das heißt die Codes C77, C78 und C79 für sekundäre Neubildungen sollten nicht verwendet werden. Die Antwort sollte also niemals "C77", "C78" oder "C79" sein.

Prompt template "0-uro"

User: Was ist der ICD-10-Code für die Diagnose "[DIAGNOSIS-LABEL]"? Antworten Sie nur kurz mit dem 3-stelligen ICD-10-Code. Wenn es sich bei der Diagnose "[DIAGNOSIS-LABEL]" um ein Prostatakarzinom handelt, antworten Sie zum Beispiel mit "C61", bei einem Urothelkarzinom der Harnblase mit "C67". Die Metastasierung muss bei der Kodierung nicht berücksichtigt werden, das heißt die Codes C77, C78 und C79 für sekundäre Neubildungen sollten nicht verwendet werden. Die Antwort sollte also niemals "C77", "C78" oder "C79" sein.

Prompt template "2-gyn-ctx"

**User:** Ihnen werden Diagnosetexte vorgelegt, zu denen Sie ICD-10-Codes zuordnen sollen. Die Frage ist jeweils: Was ist der ICD-10-Code für diese Diagnose? Antworten Sie darauf nur kurz mit dem 3-stelligen ICD-10-Code. Die Metastasierung muss bei der Kodierung nicht berücksichtigt werden, das heißt die Codes C77, C78 und C79 für sekundäre Neubildungen sollten nicht verwendet werden. Die Antwort sollte also niemals "C77", "C78" oder "C79" sein.
**Assistant:** Ich werde auf die folgenden Diagnosetexte kurz mit dem ICD-10-Code antworten.
**User:** Was ist der ICD-10-Code für diese Diagnose?<br>Diagnose: Metastasiertes Mammakarzinom
**Assistant:** C50
**User:** Was ist der 3-stellige ICD-10-Code für diese Diagnose?<br>Diagnose: Ovarialkarzinom
**Assistant:** C56
**User:** Was ist der 3-stellige ICD-10-Code für diese Diagnose?<br>Diagnose: "[DIAGNOSIS-LABEL]". Die Diagnose stammt aus folgendem längeren Text: [SNIPPET-TEXT]

**Prompt template "2-gyn"**
**User:** Ihnen werden Diagnosetexte vorgelegt, zu denen Sie ICD-10-Codes zuordnen sollen. Die Frage ist jeweils: Was ist der ICD-10-Code für diese Diagnose? Antworten Sie darauf nur kurz mit dem 3-stelligen ICD-10-Code. Die Metastasierung muss bei der Kodierung nicht berücksichtigt werden, das heißt die Codes C77, C78 und C79 für sekundäre Neubildungen sollten nicht verwendet werden. Die Antwort sollte also niemals "C77", "C78" oder "C79" sein.
**Assistant:** Ich werde auf die folgenden Diagnosetexte kurz mit dem ICD-10-Code antworten.
**User:** Was ist der ICD-10-Code für diese Diagnose?<br>Diagnose: Metastasiertes Mammakarzinom
**Assistant:** C50

User: Was ist der 3-stellige ICD-10-Code für diese Diagnose?
Diagnose: Ovarialkarzinom

Assistant: C56

User: Was ist der 3-stellige ICD-10-Code für diese Diagnose?
Diagnose: "[DIAGNOSIS-LABEL]".

Prompt template "2-uro-ctx"

User: Ihnen werden Diagnosetexte vorgelegt, zu denen Sie ICD-10-Codes zuordnen sollen. Die Frage ist jeweils: Was ist der ICD-10-Code für diese Diagnose? Antworten Sie darauf nur kurz mit dem 3-stelligen ICD-10-Code. Die Metastasierung muss bei der Kodierung nicht berücksichtigt werden, das heißt die Codes C77, C78 und C79 für sekundäre Neubildungen sollten nicht verwendet werden. Die Antwort sollte also niemals "C77", "C78" oder "C79" sein.

Assistant: Ich werde auf die folgenden Diagnosetexte kurz mit dem ICD-10-Code antworten.

User: Was ist der ICD-10-Code für diese Diagnose?
Diagnose: Metastasiertes Prostatakarzinom

Assistant: C61

User: Was ist der 3-stellige ICD-10-Code für diese Diagnose?
Diagnose: Urothelkarzinom der Harnblase

Assistant: C67

User: Was ist der 3-stellige ICD-10-Code für diese Diagnose?
Diagnose: "[DIAGNOSIS-LABEL]". Die Diagnose stammt aus folgendem längeren Text: [SNIPPET-TEXT]

Prompt template "2-uro"

User: Ihnen werden Diagnosetexte vorgelegt, zu denen Sie ICD-10-Codes zuordnen sollen. Die Frage ist jeweils: Was ist der ICD-10-Code für diese Diagnose? Antworten Sie darauf nur kurz mit dem 3-stelligen ICD-10-Code. Die Metastasierung muss bei der Kodierung nicht

berücksichtigt werden, das heißt die Codes C77, C78 und C79 für sekundäre Neubildungen sollten nicht verwendet werden. Die Antwort sollte also niemals "C77", "C78" oder "C79" sein.

Assistant: Ich werde auf die folgenden Diagnosetexte kurz mit dem ICD-10-Code antworten.

User: Was ist der ICD-10-Code für diese Diagnose?
Diagnose: Metastasiertes Prostatakarzinom

Assistant: C61

User: Was ist der 3-stellige ICD-10-Code für diese Diagnose?
Diagnose: Urothelkarzinom der Harnblase

Assistant: C67

User: Was ist der 3-stellige ICD-10-Code für diese Diagnose?
Diagnose: "[DIAGNOSIS-LABEL]".

Prompt templates for Step 3

Prompt template "0-dates"

User: [Diagnosetext]:
[SNIPPET-TEXT]
[Ende des Diagnosetexts]
Sie haben eine Diagnose im Diagnosetext identifiziert: "[DIAGNOSIS-LABEL]" Finden Sie eine Datumsangabe zur Erstdiagnose (ED) dieser Tumorerkankung im Text? Die Datumsangabe kann tagesgenau, monatsgenau oder nur eine Jahresangabe sein. Antworten Sie kurz mit dem Datum, wie es im Text angegeben ist. Achten Sie darauf, dass Sie nur ein Datum angeben, wenn es sich eindeutig auf die Erstdiagnose des Tumors bezieht. Datumsangaben zu Rezidiven, Metastasen oder zur Therapie des Tumors sind hier nicht relevant. Wenn es kein passendes Erstdiagnosedatum im Text gibt, antworten Sie mit "Nein".
Folgende Datumsangaben sind im Text zu finden: [DATES]. Ist eine der Datumsangaben das Datum der Erstdiagnose zur Tumordiagnose "[DIAGNOSIS-LABEL]"?

Prompt template "0-verify"

User: [Diagnosetext]:
[SNIPPET-TEXT]
[Ende des Diagnosetexts]
Im Diagnosetext haben Sie die Diagnose "[DIAGNOSIS-LABEL]" identifiziert. Zudem ist im Diagnosetext folgende Datumsangabe zu finden: 2002. Ist dies das Datum der Erstdiagnose der Tumorerkrankung "[DIAGNOSIS-LABEL]"? Antworten Sie mit "Ja" oder "Nein". Das Erstdiagnosedatum wird oft mit der Abkürzung "ED" vermerkt. Dann ist es klar, dass es sich um das Erstdiagnosedatum handelt. Es kann auch sein, dass das Datum ohne die Angabe "ED" angegeben ist, aber ein klarer Bezug zum ersten Diagnose der Tumorerkankung gegeben ist. In diesem Fall antworten Sie mit "Ja". Achten Sie aber darauf, dass Sie nur dann mit "Ja" antworten, wenn das Datum "[DATE]" sich eindeutig auf die Erstdiagnose zur Tumordiagnose "[DIAGNOSIS-LABEL]" bezieht. Datumsangaben, die sich ausschließlich auf Rezidive, Metastasen die Therapie des Tumors beziehen, sind hier nicht relevant. Wenn das Datum "[DATE]" sich nicht klar auf die Zeit (Tag, Monat oder Jahr) der Erstdiagnose bezieht, antworten Sie mit "Nein".

Prompt template "0"

User: [Diagnosetext]:
[SNIPPET-TEXT]
[Ende des Diagnosetexts]
Sie haben eine Diagnose im Diagnosetext identifiziert: "[DIAGNOSIS-LABEL]" Finden Sie eine Datumsangabe zur Erstdiagnose (ED) dieser Tumorerkankung im Text? Die Datumsangabe kann tagesgenau, monatsgenau oder nur eine Jahresangabe sein. Antworten Sie kurz mit dem Datum, wie es im Text angegeben ist. Achten Sie darauf, dass Sie nur ein Datum angeben, wenn es sich eindeutig auf die Erstdiagnose des Tumors bezieht. Datumsangaben zu Rezidiven, Metastasen oder zur Therapie des Tumors sind hier nicht relevant. Wenn es kein passendes Erstdiagnosedatum im Text gibt, antworten Sie mit "Nein".

Prompt template "3-dates"

User: Finden Sie eine Datumsangabe zur Erstdiagnose (ED) einer gegebenen Tumorerkankung? Die Datumsangabe kann tagesgenau, monatsgenau oder nur eine Jahresangabe sein. Antworten Sie kurz mit dem Datum, wie es im Text angegeben ist. Achten Sie darauf, dass Sie nur ein Datum angeben, wenn es sich eindeutig auf die Erstdiagnose des Tumors bezieht. Datumsangaben zu Rezidiven, Metastasen oder zur Therapie des Tumors sind hier nicht relevant. Wenn es kein passendes Erstdiagnosedatum im Text gibt, antworten Sie mit "Nein".

Assistant: OK, geben Sie mir die Texte und Diagnosen. Ich werde mit dem Datum antworten, wie es im Text angegeben ist, wenn ich ein Erstdiagnosedatum zur Diagnose finde. Wenn es kein Datum dazu gibt, antworte ich mit "Nein".

User: [Diagnosetext]:
Z.n. Prostatakarzinom ED 1999
Z.n. Nierentransplantation 1980 links
[Ende des Diagnosetexts]
Im Diagnosetext haben Sie die Diagnose "Prostatakarzinom" identifiziert. Folgende Datumsangaben sind im Text zu finden: 1999, 1980. Ist eine der Datumsangaben das Datum der Erstdiagnose zur Tumordiagnose "Prostatakarzinom"? Antworten Sie mit dem Datum, wenn es die Zeit der Erstdiagnose der Tumordiagnose "Prostatakarzinom" beschreibt, oder mit "Nein".

Assistant: 1999

User: [Diagnosetext]:
- TUR-B-NR mit dem Nachweis eines Urothelkarzinoms der Harnblase pT2G3
- Prostatakarzinom ED 07/2017
[Ende des Diagnosetexts]
Im Diagnosetext haben Sie die Diagnose "Urothelkarzinom" identifiziert. Im Diagnosetext ist folgende Datumsangabe zu finden: 07/2017. Ist dies das Datum der Erstdiagnose zur Tumordiagnose "Urothelkarzinom"? Antworten Sie mit dem Datum, wenn es die Zeit der Erstdiagnose der Tumordiagnose "Urothelkarzinom" beschreibt, oder mit "Nein".

Assistant: Nein (Es gibt kein Datum zum Urothelkarzinom, nur eines für das Prostatakarzinom)

User: [Diagnosetext]:
09/2011: Invasives lobuläres Mammakarzinom links, Stadium II
10/2011: Brusterhaltende Therapie (BET)
11/2011: Adjuvante Strahlentherapie, abgeschlossen 12/2011
[Ende des Diagnosetexts]
Im Diagnosetext haben Sie die Diagnose "Mammakarzinom" identifiziert. Folgende Datumsangaben sind im Text zu finden: 09/2011, 10/2011, 11/2011, 12/2011. Ist eine der Datumsangaben das Datum der Erstdiagnose zur Tumordiagnose "Mammakarzinom"? Antworten Sie mit dem Datum, wenn es die Zeit der Erstdiagnose der Tumordiagnose "Mammakarzinom" beschreibt, oder mit "Nein".

Assistant: 09/2011

User: [Diagnosetext]:
[SNIPPET-TEXT]
[Ende des Diagnosetexts]
Im Diagnosetext haben Sie die Diagnose "[DIAGNOSIS-LABEL]" identifiziert. Folgende Datumsangaben sind im Text zu finden: [DATES]. Ist eine der Datumsangaben das Datum der Erstdiagnose zur Tumordiagnose "[DIAGNOSIS-LABEL]"? Antworten Sie mit dem Datum, wenn es die Zeit der Erstdiagnose der Tumordiagnose "[DIAGNOSIS-LABEL]" beschreibt, oder mit "Nein".

Prompt template "3"

User: Finden Sie eine Datumsangabe zur Erstdiagnose (ED) einer gegebenen Tumorerkankung? Die Datumsangabe kann tagesgenau, monatsgenau oder nur eine Jahresangabe sein. Antworten Sie kurz mit dem Datum, wie es im Text angegeben ist. Achten Sie darauf, dass Sie nur ein Datum angeben, wenn es sich eindeutig auf die Erstdiagnose des Tumors bezieht. Datumsangaben zu Rezidiven, Metastasen oder zur Therapie des Tumors sind hier nicht relevant. Wenn es kein passendes Erstdiagnosedatum im Text gibt, antworten Sie mit "Nein".

Assistant: OK, geben Sie mir die Texte und Diagnosen. Ich werde mit dem Datum antworten, wie es im Text angegeben ist, wenn ich ein Erstdiagnosedatum zur Diagnose finde. Wenn es kein Datum dazu gibt, antworte ich mit "Nein".

User: Gibt es ein Erstdiagnosedatum zur Diagnose "Prostatakarzinom" in folgendem Text?
Text:
Z.n. Prostatakarzinom ED 1999
Z.n. Nierentransplantation 1980 links

Assistant: 1999

User: Gibt es ein Erstdiagnosedatum zur Diagnose "Urothelkarzinom" in folgendem Text?
Text:
- TUR-B-NR mit dem Nachweis eines Urothelkarzinoms der Harnblase pT2G3
- Prostatakarzinom ED 07/2017

Assistant: Nein (Es gibt kein Datum zum Urothelkarzinom, nur eines für das Prostatakarzinom.)

User: Gibt es ein Erstdiagnosedatum zur Diagnose "Mammakarzinom" in folgendem Text?
Text:
09/2011: Invasives lobuläres Mammakarzinom links, Stadium II
10/2011: Durchführung einer brusterhaltenden Therapie (BET)
11/2011: Beginn der adjuvanten Strahlentherapie, abgeschlossen 12/2011

Assistant: 09/2011

User: Gibt es ein Erstdiagnosedatum zur Diagnose "[DIAGNOSIS-LABEL]" in folgendem Text?
Text:
[SNIPPET-TEXT]

Prompt template "4-verify"

User: Sie erhalten Diagnosetexte und müssen bestimmen, ob ein bestimmtes Datum das Erstdiagnosedatum einer Tumorerkrankung ist. Antworten Sie mit "Ja" oder "Nein". Das Erstdiagnosedatum wird oft mit der Abkürzung "ED" vermerkt. Dann ist es klar, dass es sich um das Erstdiagnosedatum handelt. Es kann auch sein, dass das Datum ohne die Angabe "ED" angegeben ist, aber ein klarer Bezug zur ersten Diagnose der Tumorerkankung gegeben ist. In diesem Fall antworten Sie mit "Ja". Achten Sie aber darauf, dass Sie nur dann mit "Ja" antworten, wenn das genannte Datum sich eindeutig auf die Erstdiagnose zur Tumordiagnose bezieht. Datumsangaben, die sich ausschließlich auf Rezidive, Metastasen die Therapie des Tumors beziehen, sind hier nicht relevant. Wenn das Datum sich nicht klar auf die Zeit (Tag, Monat oder Jahr) der Erstdiagnose bezieht, antworten Sie mit "Nein".

Assistant: OK, geben Sie mir die Texte und die Datumsangaben. Ich werde mit "Ja" antworten, wenn das Datum die Erstdiagnosedatum zur Diagnose beschreibt, ansonsten antworte ich mit "Nein".

User: Ist "1999" die Datumsangabe für die Erstdiagnose der Tumorerkrankung "Prostatakarzinom" im Text? Text:
Z.n. Prostatakarzinom ED 1999
Z.n. Nierentransplantation 1980 links

Assistant: Ja. 1999 ist eindeutig als Erstdiagnosedatum (ED) bei der Diagnose "Prostatakarzinom" angegeben.

User: Ist "07/2017" die Datumsangabe für die Erstdiagnose der Tumorerkrankung "Urothelkarzinom" im Text? Text:

- TUR-B-NR mit dem Nachweis eines Urothelkarzinoms der Harnblase pT2G3<br>- Prostatakarzinom ED 07/2017
**Assistant:** Nein. Es gibt kein Datum zum Urothelkarzinom, die Datumsangabe "07/2017" bezieht sich auf das Prostatakarzinom.
**User:** Ist "10/2011" die Datumsangabe für die Erstdiagnose der Tumorerkrankung "Mammakarzinonm" im Text? Text:<br>09/2011: Invasives lobuläres Mammakarzinom links, Stadium II<br>10/2011: Durchführung einer brusterhaltenden Therapie (BET)<br>11/2011: Beginn der adjuvanten Strahlentherapie, abgeschlossen 12/2011
**Assistant:** Nein. Die Datumsangabe "10/2011" bezieht sich auf eine Therapie des Mammakarzinoms, nicht auf die Erstdiagnose der Tumorerkrankung.
**User:** Ist "09/2011" die Datumsangabe für die Erstdiagnose der Tumorerkrankung "Mammakarzinom" im Text? Text:<br>09/2011: Invasives lobuläres Mammakarzinom links, Stadium II<br>10/2011: Durchführung einer brusterhaltenden Therapie (BET)<br>11/2011: Beginn der adjuvanten Strahlentherapie, abgeschlossen 12/2011
**Assistant:** Ja. Hier ist anzunehmen, dass sich die Datumsangabe auf die Erstdiagnose des Mammakarzinoms bezieht, auch wenn die Angabe nicht explizit mit "ED" gkennzeichnet ist.
**User:** Ist "[DATE]" die Datumsangabe für die Erstdiagnose der Tumorerkrankung "[DIAGNOSIS-LABEL]" im Text? Text:<br>[SNIPPET-TEXT]
